# Psychofarmacological approach for Binge- eating disorders

**DOI:** 10.1192/j.eurpsy.2022.596

**Published:** 2022-09-01

**Authors:** V. Muñoz Martinez, L. Beato-Fernández, E. Segura-Escobar

**Affiliations:** 1 Hospital General Universitario de Ciudad Real, Adolescents Inpatient Unit., Ciudad Real, Spain; 2 Hospital General Universitario de Ciudad Real, Adolescente Inpatient Unit., Ciudad Real, Spain; 3 Hospital General Universitario de Ciudad Real, Chief Of Psychiatry, Ciudad Real, Spain

**Keywords:** binge eating disorder, Lisdexanfetamine, Fluoxetine, eating disorder

## Abstract

**Introduction:**

Binge-eating disorder (BED), is one of the most common eating disorder. Treatment aims to reduce binge-eating frequency and disordered eating–related cognitions, improve metabolic health and weight, and regulate mood (in patients with coexisting depression or anxiety)

**Objectives:**

The aim of this study was to examine the efficacy of lisdexamfetamine dimesylate in a simple of 50 women with a binge eating disorder diagnosis compare with selective serotonin reuptake inhibitor

**Methods:**

Two groups were made, one with lisdexamfetamine and the other with selective serotonin reuptake inhibitor (fluoxetine). 20 women were in each group (total n=40). The doses depend of the binge symptoms and rates were from 30 to 70md/day for lisdexamfetamine and for fluoxetine the doses were from 20 to 60mg/day.

**Results:**

Binge behaviors decreased with a 50mg/day dose of lisdexamfetamine. The 70mg/day doses present also less binge behaviors but also more adverse events. The 30mg/day doses did not decrease binge-eating behaviors.

**Conclusions:**

Lisdexamfetamine is the first pharmacological agent to receive FDA approval for use in adults with moderate to severe binge eating disorder. This study supports further assessment of lisdexamfetamine as a treatment option for decreasing binge eating behavior and also symptoms associated such as anxiety and obsessive and compulsive features in adults.Increased efficacy with increasing dosages of lisdexamfetamine suggests a dose-response relationship until 50mg/day. Women with a dose of 50mg/day of lisdexamfetamine report less adverse event, more adherence to treatment and improve their eating behaviors.

**Disclosure:**

No significant relationships.

